# Kombucha: Challenges for Health and Mental Health

**DOI:** 10.3390/foods12183378

**Published:** 2023-09-08

**Authors:** Patrícia Batista, Maria Rodrigues Penas, Catarina Vila-Real, Manuela Pintado, Patrícia Oliveira-Silva

**Affiliations:** 1 Research Centre for Human Development, Human Neurobehavioural Laboratory, Universidade Católica Portuguesa, Rua de Diogo Botelho, 1327, 4169-005 Porto, Portugal; mariarodriguespenas@gmail.com (M.R.P.); posilva@ucp.pt (P.O.-S.); 2 CBQF—Centro de Biotecnologia e Química Fina, Laboratório Associado, Escola Superior de Biotecnologia, Universidade Católica Portuguesa, Rua de Diogo Botelho, 1327, 4169-005 Porto, Portugal; cvreal@ucp.pt (C.V.-R.); mpintado@ucp.pt (M.P.)

**Keywords:** Kombucha, probiotics, mental health, mental diseases, microbiota

## Abstract

Background: Increasing research into probiotics is showing potential benefits for health in general and mental health in particular. Kombucha is a recent beverage and can be considered a probiotic drink, but little is known about its effects on physical and mental health. This product is experiencing growth in the market; however, there are no scientific results to support its potential for physical and mental health. Aim: This review article aims to draw attention to this issue and to highlight the lack of studies in this area. Key findings and conclusions: The lack of legislation for the correct marketing of this product may also constrain clinical studies. However, clinical studies are of utmost importance for an in-depth understanding of the effects of this product on the human body. More research is needed, not only to better understand the impact of Kombucha on the human body, but also to ensure the application of regulatory guidelines for its production and marketing and enable its safe and effective consumption.

## 1. Introduction

Nowadays people are increasingly concerned about their own health. People want to become healthier, and the adoption of healthier lifestyles is necessary to improve quality of life and to reduce the numerous pathologies and comorbidities associated with nutrient inadequacy or poor diet choices [1]. People are concerned about their body, their mind and their health, and so their search for healthy foods or specific foods that improve health, such as functional foods, has been increasing [2,3,4]. Functional foods have been linked to improved health and greater quality of life. These products are defined as dietary products (processed or natural foods) that when consumed regularly in a balanced and diversified diet, improve both the mental and physical state of health and/or reduce the risk of disease [5,6]. These functional foods can be classified from the product point of view as follows: fortified (with additional nutrients), enriched (new nutrients or components), altered (substance(s) replacement by other(s) with beneficial effects), and/or enhanced products (new feed composition, genetic manipulation) [5]. Thus, in addition to their nutritional value as conventional foods, these foods present potential to enhance health and can reduce the risk of developing non-communicable diseases, such as cancer, diabetes, cardiovascular diseases, and mental illnesses [7]. Although there is not yet a worldwide acceptable definition for functional foods, a definition that may become integrative is that of the Functional Food Centre (FFC). The FFC defines functional food as ‘natural or processed foods that contain known or unknown biologically-active compounds which, in defined, effective, and non-toxic amounts, provide a clinically proven and documented health benefit for the prevention, management or treatment of a chronic disease’ [8].

According to the literature, some of the main components of functional foods are polyunsaturated fatty acids, probiotics/prebiotics/synbiotics, and antioxidants [6]. The current research highlights the role of probiotics in functional foods and their impacts on mental health. The development of functional foods and beverages with probiotics has shown a marked increase over the last years, and the consumer and market demand for these products is increasing all over the world [9]. According to *Fortune Business Insights* the global market was valued at USD 48 billion in 2019 and it is projected to reach USD 94 billion by 2027 [10]. Probiotics have been in the spotlight lately mainly due to their impact on the modulation of the gut microbiota and to other irrefutable health benefits, such as neurological and immunological effects [11,12]. Currently, it is known that physical and mental health are controlled by the brain and one’s environment, but recent discoveries have placed a special role on the gut microbiota [13,14]. Recent findings show that there is a relation between what we consume and our mental health, more precisely, between the brain and the gut [13], which means that both emotional and behavioral processing are affected not only by the brain and the environment, but also by the gut microbiota [13,15].

The consumption of probiotics is increasing, and there is a great diversity of probiotic products on the market [11,12]. These products are displayed in several forms such as supplements (e.g., powder, capsules), food (e.g., yoghurts, cheese, butter, soy products, fermented vegetables, processed food, among others), and beverages [16].

### 1.1. The Kombucha Beverage

**Figure 1:**
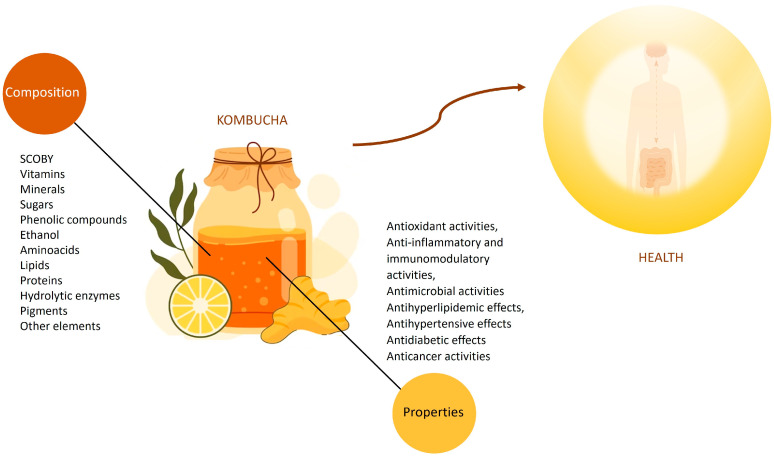
Composition and properties of Kombucha beverage.

One popular beverage that has been gaining visibility is Kombucha. Kombucha is a traditional Chinese beverage made of sweetened tea (mainly black or green; however, other variants of tea and also other ingredients, such as cereals, milk, and even mushrooms, may be used) fermented by a specific combination of probiotic microorganisms. Kombucha mainly comprises tea polyphenols, amino acids, vitamins, minerals, organic acids, sugars, and probiotic microorganisms. This is a functional beverage that has a high amount of plant extracts and metabolites resulting from the metabolism of the symbiotic culture of bacteria (mainly *Lactobacillus* and *Bifidobacterium* species) and yeasts (mainly *Saccharomyces* species), known as SCOBY, [17,18]. According to the literature, Kombucha has been shown to have physiological benefits, such as anti-inflammatory, antibacterial, and antioxidant activities, alongside other properties. Consequently, it qualifies as a functional beverage [19,20,21] (see Figure 1).

Kombucha is very rich in various bioactive compounds, including acetic and gluconic acid, complex B vitamins, minerals, amino acids, and polyphenols, that have been recognized as having health benefits, such as anti-inflammatory, antioxidant, antidiabetic, and antimicrobial activity [22]. Different studies have shown that the type and amount of these bioactive compounds are associated not only with the properties of the black or green tea used (other materials can also be used, such as cereals, milk, and even mushrooms) but also with the fermentation conditions, namely, the type of microorganisms and their interactions, time and temperature [22,23].

The literature reports the impact of the biological properties of Kombucha on health and several disorders, such as inflammatory diseases, arthritis, allergies, cardiovascular diseases, and cancer [24]. Some studies highlight that Kombucha’s composition impacts hypocholesterolemic activity, as well as anti-hypertensive and antidiabetic activities, by inhibiting the enzymes angiotensin-converting enzyme, α-amylase and β-glucosidase, respectively [24].

On the other hand, some studies have correlated the consumption of probiotics with reductions in anxiety and depression [25,26,27], and a positive impact on cognitive and emotional functions [28]. However, it is not well established how its consumption can affect the human “psyche”. It is known that probiotics modulate the intestinal microbiota which subsequently influence brain responses, such as neurotransmitter biosynthesis and the emotional state, through the gut–brain axis [29].

Thus, the present review focuses on the role of probiotic Kombucha in mental health and associated diseases and intends to draw attention to this thematic, summarizing the latest publications in this field and aiming to understand its potential impact on health.

### 1.2. Mental Health via Gut–Brain Axis

The literature contains increasing evidence that corroborates the mutual influence between the brain and the gut microbiota [25,30]. The gut microbiota–brain axis is a bidirectional structure that connects these two organic systems and has effects on the modulation of human organic and psychological functions [30]. For example, substances released by the gut microbiota can reach the brain via the circulatory pathway. On the other hand, the brain may also be influencing the gut microbiota by neuronal and endocrine pathways. The literature reports that this communication occurs through three main pathways: the autonomic nervous system (including the enteric nervous system and vagus nerve), immune system and neuroendocrine system [14,31,32].

The autonomic nervous system (ANS) is a neural network that comprises the sympathetic and parasympathetic systems. This network in connection with hypothalamic-pituitary–adrenal axis establishes the communication between the brain and the gut [14]. The ANS is responsible for controlling physiological homeostasis without conscious effort, namely the gastrointestinal function. The ANS in connection with neuronal (e.g., vagus nerve) and neuroendocrine signaling can induce changes in the gastrointestinal autonomic activation, which can be triggered by interoceptive afferent feedback or by cognitive and emotional efferent modulation [14,32,33]. On the other hand, the ANS controls several gastrointestinal functions, such as gut permeability, fluids production, and the mucosal immune response, and it can be modulated by gut microbiota metabolites like short-chain fatty acids (SCFAs) and lipopolysaccharides [14,32]. Therefore, the ANS provides the gut with the most direct neurological response available; however, the intestinal microbiota can interact with gut ANS synapses, by microbiota-derived neuromodulatory metabolites (e.g., serotonin (SER), gamma-aminobutyric acid (GABA), catecholamines). This process explains the microbiota–gut–brain axis bidirectional communication. Thus, changes in the gut microbiota can influence brain functions (e.g., cognitive, memory, emotion, decision-making) and behaviors (e.g., mood) [14,32,34].

The role of the immune system’s pathway role in gut–brain signaling is growing [35]. It is known that neuroinflammation increases the odds of developing psychiatric disorders, such as autism spectrum disorders, epilepsy, Alzheimer’s disease, Parkinson’s disease and cerebrovascular diseases [14,35,36]. The literature reports that various gut metabolites, including SCFAs (e.g., butyrate, propionate, acetate), bacteriocins and neuromodulators (e.g., glutamate), seem to activate the immune system and so affect and regulate cytokine secretion and microglial activation [14]. Thereby, cytokines, neurotransmitters, neuropeptides, SCFAs and other microbiota metabolites can pass through the blood and lymphatic systems and be in constant communication with both the brain and the gut [14,32,37].

Finally, the other most studied pathway in this axis communication is the neuroendocrine pathway. As with the immune system, gut metabolites can enter into the systemic circulation, activate the hypothalamic–pituitary–adrenal axis, and modify the secretion of gut hormones and neurotransmitters, and consequently regulate body metabolism functions [14,33]. The literature shows that homeostatic deregulation can be caused by mental disorders, such as stress, anxiety, or depression [35]. For example, in stress disorders, there is a release of the adrenocorticotrophic hormone by the pituitary gland and cortisol by the adrenal cortex, which seems to be associated with the neuroimmune response and cytokine release, leading to an increase in gastrointestinal permeability [14,38,39].

Such a complex mechanism that involves not only internal but also external factors, such as stress, diet, exercise, and the environment, has interested researchers and, in consequence, much research has been conducted to understand these interactions [38].

### 1.3. Probiotics/Kombucha Impact on Mental Health

As mentioned above, probiotics are functional components. According to the World Health Organization (2001), probiotics are live microorganisms that when incorporated into the daily diet in adequate amounts, confer physical and mental health benefits on the host [40,41]. Their therapeutic potential has been explored in several pathologies, such as obesity, cancer, inflammatory bowel disease, diabetes, arthritis, and mental disorders. Therefore, the impacts of probiotic products on the gut–brain relation and how altered gut microbiota affects mental illness (e.g., depression, anxiety, bipolar disorder, schizophrenia) and its prevention and/or treatment have been studied [28].

Several microorganisms (bacteria and yeasts) have been reported as probiotics, namely, specific probiotic strains of the following genera: *Bifidobacterium*, *Lactobacillus*, *Propionibacterium*, *Peptostreptococcus*, *Pediococcus*, *Leuconostoc*, *Enterococcus*, *Streptococcus*, *Bacillus*, *Bacteroides*, *Akkermansia*, and *Saccharomuyces* [40,42]. These probiotic strains within the abovementioned genera are known for boosting the immune system and balancing the intestinal microbiota [12] and may act as prevention or therapeutics for cardiovascular diseases and cancer, among others [43]. Specifically in regard to the positive impacts of probiotics on brain disorders (e.g., multiple sclerosis, amyotrophic lateral sclerosis, Alzheimer and Parkinson’s disease, autism, stress, and anxiety), the literature reports the potential of several species and strains belonging to the following genera: *Actinobacteria*, *Aelobaculum*, *Alistipes*, *Allobaculum*, *Anaerotruncus*, *Akkermansia*, *Bacteroidetes*, *Bacteroidesfragilis*, *Bascteroidesvulgatu*, *Blautia*, *Bifidobacterium*, *Bilophila*, *Butyrivibriofibrisolvens*, *Collinsella*, *Clostridium*, *Coprococcus*, *Corynebacterium*, *Desulfovibrio*, *Dialister*, *Disulfovibironacease*, *Dorea Enterobacteriaceae*, *Faecalibacterium*, *Firmicutes*, *Methanobrevibacter*, *Oscillibacter*, *Parabacteroidsdistasonis*, *Parabacteroides*, *Peptococcus*, *Prevotellaceae*, *Proteobacteria*, *Ralstonia*, *Rikenellaceae*, *Roseburia*, *Ruminococcus*, *Sutterellaceae*, *Tenericutes*, *Veillonella*, and *Verrucomicrobia* [42,44].

The literature suggests that the consumption of these probiotic foods and supplements may enhance cognitive function. With that, the reduction in negative neurologic or neuropsychiatric conditions, such as depression, anxiety, stress related symptoms, emotional deregulation, some neurodegenerative diseases (Alzheimer’s, and Parkinson’s disease) can even help with antisocial and aberrant behaviors associated with autism disorder [29,45,46,47].

Although the literature reports the advantages of probiotics and probiotic food in mental health, less is known about the role of these foods in the gut–brain relation, their importance in CNS function, and in neurodevelopment. Some animal and human studies have reported that probiotics contribute to neuronal modulations through the release of their metabolites, such as GABA, Serotonin, SCFAs, Glutamate, and others. For example, Hur and collaborators (2022) reported that in their animal study, treatment with probiotics relieved hyperactivation of the hypothalamic–pituitary–adrenal axis, which decreased cortisol levels and reduced anxiety and depressive symptoms [34]. The probiotics’ anti-inflammatory effect modulates the proinflammatory cytokines and can potentially decrease depressive symptoms. Probiotics have also been shown to alleviate depressive mood in humans; however, studies in humans are still scarce [34]. Although other animal studies have been conducted [48], further studies, both in animals and humans, are needed to validate the beneficial effects of probiotic supplementation on mental health conditions (depression, anxiety and other brain functions).

One probiotic food that has gained particular attention is Kombucha, and the present review aims to investigate the impacts of this specific product on mental health. Regarding the impact of Kombucha on mental health, several studies have reported the health benefits of this functional beverage, and some of its beneficial properties include antioxidant, anti-inflammatory [19,20], and antimicrobial activities [49], as well as improving immunologic and liver functions, particularly the hepaprotective effect [20,50,51]. Although the literature refers to the possible effects of Kombucha on mental health, there are few studies to support these claims, and hence the need for this review.

## 2. Methodology

This review intended to analyze the relationship between “Kombucha and mental health”. However, due to the lack of studies in the area, the search was refined and a review on “Kombucha and health” was included to identify information on in vitro, in vivo and human assays. To this end, a systematic search was performed using Pubmed, Science Direct and Web of Science databases from 2 June to 29 July 2023. Several descriptors were used: “Kombucha” AND “health” AND (“in vitro” OR “in vivo” OR “human assays”) (Figure 1). The screening of the articles was conducted by applying the following eligibility criteria:

(a) Inclusion: research articles, clinical assays, publications written in English.
(b) Exclusion: outside the scope of the subject, other types of publications (reviews, comments, editorials, discussions, correspondence, letters, short communications), publications in languages other than English, and studies whose full texts were not available.

This review was conducted using the PRISMA criteria for preferred reporting items within systematic reviews and meta-analyses (PRISMA) [52,53]. The collected information was compiled and analyzed regarding the year of publication, authors, sample, country, methodology/type of study, results, conclusions, and research aims. The bibliographic references were compiled through the computer program EndNote bibliographic referencing.

## 3. Results

**Figure 2:**
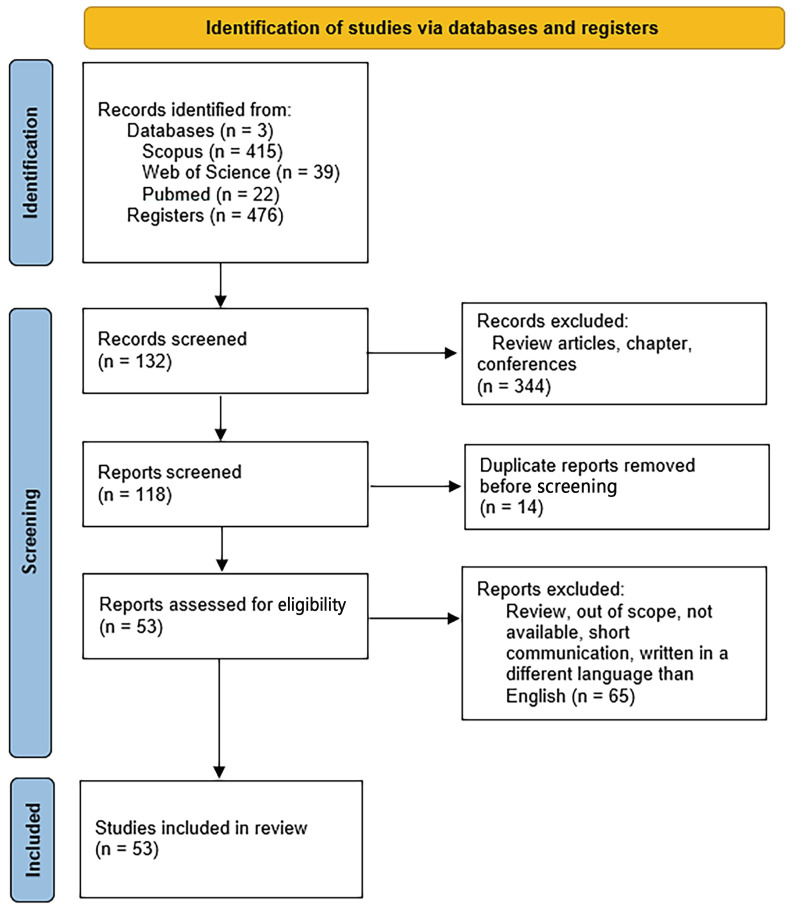
PRISMA flow diagram showing research methodology: “Kombucha” AND “mental health” AND (“in vitro” OR “in vivo” OR “human assays”).

This systematic review identified 476 scientific articles published in international journals indexed to the digital databases used in this search. After screening, duplicate publications and research that fitted the exclusion criteria were removed. Thus, after the eligible criteria were applied, fifty-three publications met the defined inclusion criteria, as shown in the PRISMA flow diagram presented in Figure 2. A summary of the most important characteristics of these articles is presented in Table 1.

It should be noted that no specific studies evaluated the impact of Kombucha on mental health (brain impact, cognitive functions, or brain disorders). Most studies reported the characteristics of the Kombucha beverage, highlighting the physicochemical and biological properties of the product. In vitro and in vivo studies with animal models were included, as well as studies of new products (incorporation of new ingredients) and new processing methods (e.g., fermentation times).

Considering the abovementioned collected data, the lack of clinical assays related to Kombucha is evident (Table 1). Human assays are scarce, and in vivo assays, although they exist, are not yet fully conclusive. Most of the studies that aimed to identify and evaluate the properties of this functional beverage (some studies reported on commercialized Kombucha already in the market and others on Kombucha still in the development process) focused on the physiochemical and biological characterization, using in vitro assays. Based on the analysis of the included studies, it is also worth highlighting the lack of information about the SCOBY used, which is why its inclusion in Table 1 was not justified.

Regarding the studies presented, most of the time, it is difficult to understand the pathology with which they are associated. However, some studies highlight using Kombucha treatments mainly for oncological diseases, diabetes, and cardiac diseases. There is a need for more practical studies about the impact of the consumption of Kombucha on the brain and brain-related diseases.

## 4. Discussion

This systematic review was designed to provide insights into the impact of Kombucha consumption on health in general, and its effects on mental health or brain disorders, in particular. Due to the appeal of Kombucha among consumers, and its potential higher consumption, together with the lack of evidence about its impact on health, specifically in mental health, careful in-depth study of these products is of utmost importance.

According to our results, it is clear there is an absence of scientific evidence/studies that can confirm the veracity of the commonly misused expression ‘the beneficial impact of Kombucha in mental health’. In consequence, readers should be cautious, not only when accessing online information, but also when it comes to Kombucha consumption.

Despite the ambiguous discussions about Kombucha found in the literature, in the obtained data, and after using several descriptors, we were only able to identify two articles with experimental designs related to this thematic and these articles were without a clear focus. Permatasari and collaborators reported the potential of Kombucha in anti-ageing [71]. However, the authors also emphasizes that evidence is needed for determining the functional potential of Kombucha consumption in humans. On the other hand, in the research works of Barbosa and collaborators [65] and other research teams (see Table 1), study emphasis was placed on the antioxidant potential of Kombucha, highlighting also the need for further studies.

Most of the studies we found in these three credible scientific databases focused on identifying and characterizing the physicochemical and biological properties of this functional beverage. Some in vitro and in vivo studies are presented, although in vivo assays are also few, and they are not yet fully conclusive. Human assays are scarce, and there is an urgent need to better understand the impact of this specific probiotic beverage on health. However, the lack of legislation and regulatory guidelines for the production process may be one reason for the lack of research. The literature has highlighted the beneficial properties of Kombucha, but the clinical evidence is limited, especially in relation to brain disorders.

The literature has reported the role of Kombucha as a possible neuroprotective agent for neurodegenerative diseases and brain damage lesions, mainly due to its antioxidant properties that are responsible for decreases in neural cell death [24,71,106]. In the studies presented in Table 1, there is clear evidence of the antioxidant potential of this functional beverage; however, it is important to transpose these findings to clinical trials. This biological property is of great importance, since the antioxidant activity is at the basis of preventing the development of neurodegenerative diseases and other non-neurological diseases. There are strong arguments and evidence for the possible correlation between this Kombucha activity and reductions in the development of neurodegenerative diseases; nevertheless, this correlation must be tested in order to draw solid conclusions [71].

Furthermore, studies have shown that glutamate, one of the most abundant metabolites in Kombucha, is the most important excitatory neurotransmitters in the brain associated with learning, memory, and neural development [83,107]. Furthermore, glutamate with GABA, effectively reduces the symptomology of anxiety, depression, and bipolar disorders [108,109]. This information helps us to understand not only the therapeutic potential of Kombucha, but also its possible preventive effect against psychiatric and neurological disorders. However, it is quite ambitious and, in fact, misleading to say that there is a cause–effect relation given the lack of studies addressing this question. Therefore, it is of paramount importance to investigate the glutamate role in Kombucha, how it reacts with other Kombucha components and if it really has a significant impact on mental health.

In addition, the anti-inflammatory properties associated with Kombucha must also be better analyzed as there is limited scientific evidence reported by only few studies [66,83,97]. This property is essential to understand neurodegenerative diseases because in these diseases, inflammation is a complex multifactorial process involving the central nervous system.

These beneficial properties of the product are due not only to the substrates used in its composition and other elements (e.g., phenolic compounds, proteins, vitamins, etc.), but also to the composition of the applied SCOBY. The literature vaguely mentions these microorganisms, providing little information on their type and quantity. Most of the articles included in this review did not report this information, and so details about SCOBY were not included in Table 1.

In the literature, one can find several studies about probiotic strains and their impact on mental health. However, the validation of their effects should be extended to specific products. Studies in the literature report how the consumption of probiotic supplements (composed mainly of *Bifidobacterium*, *Lactobacillus* and *Actinobacteria*) as part of a therapy can significantly reduce depression symptomatology, improve cognitive functions (e.g., memory and neuroplasticity), and regulate emotional behavior. Nevertheless, it is crucial to assess specific types of probiotics [25,47,110,111,112], as well as particular food matrices, which are sources of these probiotics. Some studies indicate that treatment with probiotic supplements, containing mainly *Lactobacillus* and *Bifidobacterium*, with quantities around 2 × 10^9^ CFU/g, primarily for 8–12 weeks, can significantly reduce Alzheimer’s and other neurodegenerative disease symptoms as well as elevate the production of serotonin, dopamine, norepinephrine and GABA, thereby reducing depression symptomology [113]. A specific study showed that a probiotic treatment consisting of supplementation of 8 × 10^9^ CFU/day over 12 weeks for schizophrenia could reduce the condition’s general symptomology [114,115]. It is obvious that probiotic really do impact our health and reduce the development of certain diseases, specifically neurological ones. Still, given that Kombucha consumption is increasing as a probiotic source, more studies must be performed in order to understand how this beverage can particularly influence the improvement in neurological and psychological conditions and to determine suitable doses to treat different diseases.

Thus, information about SCOBY deserves special attention, especially concerning the creation of regulatory guidelines for the development/production and commercialization of this product to ensure its quality and safety.

Another critical factor that influences Kombucha properties concerns the manufacturing process, namely the fermentation process. This review highlighted some studies that report that the fermentation conditions and time affect the antimicrobial and antioxidant activities and phenolic, flavonoid, and bioactive compounds of Kombucha [54,84,90]. For example, Aung and collaborators showed that using higher fermentation temperatures boosts the microorganism growth, maintains important total flavonoid compounds and enhances the ɑ-amylase inhibitory activity [67].

These characterization studies are extremely important, but there is an urgent need for further studies in humans. These studies are clearly scarce but are urgent because the consumption of this product is increasing. Clinical human assays should seek to understand the mechanism of action of probiotic foods and drinks on brain function, particularly on how this interaction occurs and what results from it in terms of cerebral activity. As Kombucha is one of the probiotic beverages with potential for expansion in the market, the implications of its consumption on the human body must be more deeply investigated [116], particularly its main effects on general health, as well as brain activity. On the other hand, the lack of legislation may be an obstacle to the research into impactful novel properties of this beverage [117]. Thus, more human clinical trials need to be conducted in order to guarantee the efficacy and safety of this product’s production and consumption. Only with clinical studies will it be possible to improve knowledge about this product, clearly understand its benefits, and explore the associated risks, particularly its potential toxicity.

The data reported in this review are intended to draw attention to this issue and its importance. It is extremely important to understand the real impact of Kombucha consumption on health and mental health through clinical trials and to define national and international regulatory guidelines for the production and commercialization of this beverage in order to standardize its consumption and ensure consumer health and safety [117]. The lack of such regulations and artisanal production can compromise the quality and safety of the beverage [116]. This drink involves microbiological complexity, which must be preserved according to specific guidelines.

Some limitations in the present review should be highlighted. Important limitations were the difficulty in selecting the specific descriptors and the lack of studies in the area, especially in mental health, which hampered the review process.

## 5. Conclusions and Future Perspectives

The literature has shown a link between the consumption of probiotics and the benefits associated with mental health, although the scientific evidence about the mental health benefits of the Kombucha beverage is not clear.

Even though probiotic supplements may have positive effects on the prevention and treatment of mental disorders, more studies are needed to explore the different probiotic-rich functional products in the market.

This review intended to draw attention to the lack of up-to-date scientific evidence about the health effects of Kombucha, particularly mental health-related benefits. In particular, the neuroprotective effect so often associated with Kombucha needs to be carefully studied and its potential biological activity validated. In addition, to ensure safe consumption, it is of utmost importance to apply regulatory guidelines to its production and marketing. Also, the lack of existing legislation regarding this product may also constrain the execution of clinical trials.

## Figures and Tables

**Table 1:** Summary information of the studies included from the literature search using the descriptors “Kombucha” and “health”, and “in vitro”, “in vivo” and “human assays”.

Year Ref	Aim	Methodology				Main Results
		Kombucha Production/development		Type of study	Features	
		Kombucha source	Fermentation process			
2023						
[54]	To evaluate properties of cocoa honey-based Kombucha.	Cocoa honey	30 °C 8 days	Characterization and in vitro assay	- Total microbes; - TPC and TFC; - AOA.	The fermentation time affected the total microbe, physicochemical, in vitro antioxidant activity, and phenolic and flavonoid contents of this Kombucha.
[55]	To evaluate the bioactive properties of plant tea and the Kombucha produced.	Anatolian hawthorn Kombucha, nettle leaves Kombucha	25 °C 21 days	Characterization	- Acidity and pH; - TPC and TFC; - AOA; - AMA.	Anatolian hawthorn and nettle leaves are interesting for the production of Kombucha beverages which have distinct bioactive properties.
[56]	To investigated the application of a sustainable production model of Kombucha.	Organic Assam black tea leaves (*Camelia sinensis* L.) and locally sourced Moraiolo olive leaves (*Olea europaea* L.)	25 °C 12 days	Characterization	- Chemical analysis; - TPC; - Tannin content; - AOA; - Sensory analysis.	Olive leaves (polyphenol-rich substrate) may represent a viable strategy to valorize agro-industrial waste, reduce production costs, preserve the antioxidant properties of Kombucha and enrich its sensory profile.
[57]	To produce Kombucha using coffee infusion, and to evaluate the physicochemical profile, antioxidant, microbial, and possible toxic effects in vivo.	Arabic coffee infusion	25 °C 0, 6, 12, 15 and 21 days	Characterization	- pH and total acidity; - TSS; - RS; - Bioactive compounds analysis; - AOA and AMA; - ABA and AFA.	Kombucha producion is feasible since the changes in the physicochemical characteristics, such as reduction in the contents of total soluble solids and reducing sugars and increase in acidity expressed as acetic acid proved the metabolic activity of SCOBY. According to the pH value, coffee infusion can be considered safe from a microbiological point of view.
				In vivo assay	- n = 50 (5 groups with 10 *Galleria mellonella* larvae); - Toxicity assays.	
[58]	To evaluate the hangover relieving effect of ginseng berry Kombucha (GBK) fermented with *Saccharomyces cerevisiae* and *Gluconobacter oxydans* in in vitro and in vivo models.	Black tea infusion. Ginseng berries	30 °C 18 days	In vitro assay	- Radical scavenging activity; - Content of ginsenosides; - Cell viability assay (HepG2 cells); - mRNA expression; - Analysis of genes related to OS and alcohol metabolism; - Malondialdehyde assay.	The radical scavenging activity of GBK was increased by fermentation. In HepG2 cells in which oxidative stress was induced, GBK significantly increased the expression of antioxidant enzymes by upregulating the Nrf2/Keap1 pathway. GBK significantly reduced blood ethanol and acetaldehyde concentrations in ethanol-treated mice. GBK significantly increased the levels of alcohol-metabolizing enzymes, including alcohol dehydrogenase and acetaldehyde dehydrogenase. The behavioral assays revealed that high-dose GBK significantly ameliorated ethanol-induced behavioral changes. GBK exerted a protective effect against ethanol-induced liver damage by regulating the Nrf2/Keap1 pathway.
				In vivo assay	- n = 30 ICR mice (5 groups); - Blood ethanol and acetaldehyde concentrations; - ADH and ALDH activities in the liver tissue; - Serum alanine aminotransferase, aspartate aminotransferase, glucose, and lactate dehydrogenase levels; - Behavioral analysis following ethanol administration.	
[59]	To determine the glycemic index and insulin index responses when a standard high carbohydrate, high GI meal is consumed with a complex living Kombucha, compared with either soda water or diet soft drink.	Organic Kombucha (The Good Brew Company Pty Ltd., Victoria, Australia)	Not reported	Human assay	- Randomised, single-blinded, placebo-controlled crossover design with 11 healthy adults with normal glucose tolerance and BMI, aged between 18 and 45 years.	There wase no statistically significant difference in GI or II between the standard meal consumed with soda water or diet soft drink. In contrast, when Kombucha was consumed, there was a clinically significant reduction in GI and II compared with the meal consumed with soda water.
[60]	To carry out the physicochemical, antioxidant, and enzymatic characterization of green tea and Kombucha.	Green tea leaves Commercial Kombucha, Viva Mais^®^ brand,	24 °C 7 days	Characterization	- pH and total acidity; - TSS and TPC;. - AOA; - thiobarbituric acid reactive substances; - EIC.	Kombucha had lower pH, higher acidity, and solids content compared with green tea. TPC showed no significant difference between the beverages. Green tea and Kombucha presented significant antioxidant capacity, and significant inhibitory activity of the α-glucosidase enzyme; however, green tea presented superior inhibitory potential. Kombucha exhibited pro-oxidant activity.
2022						
[61]	To identify Kombucha’s microbial community during storage.	Organic green tea and aromatic herbs: *Aloysia citrodora* (lemon verbena), *Malva sylvestris* (mallow), *Rosa* spp. (wild rose), Mentha × piperita (peppermint)	25 °C 4 days	Characterization	- Microorganisms molecular identifcation; - Functional characterization of yeast isolates.	This Kombucha represents not only a plant-based, non-dairy fermented beverage, but also a valuable source of potentially functional yeast strains.
[62]	To develop newly formulated bread products from Kombucha tea, and to compare them with the classical bread recipe based on some instrumental and sensory quality characteristics.	Kombucha tea, made from black (KBT) and green tea (KGT)	33 °C 9 days	Characterization	- Physical characteristics; - TPC; - AOA; - Sensory analysis.	Breads enriched with both Kombuchas had higher specific volume, greater pore ratio, lower moisture content and higher mineral content than the control bread. Bread with KBT presented a dark color formed by browning reactions of phenolic compounds in the black tea. Kombuchas affected the textural characteristics of the breads: higher hardness and less elasticity were obtained. Bread enriched with KBT was liked and preferred as the best bread by panelists.
[63]	To assess the effect of Sea grapes Kombucha drink on lipase activity in vitro and the lipid profile in vivo.	Sea grapes *(Caulerpa racemosa*)	20–25 °C 12 days	In vitro assay	- Lipase inhibitory activity;	The lipase inhibitory activity of Kombucha was similar to orlistat (an inhibitor of gastrointestinal lipase approved for for obesity and dyslipidemia treatments). Kombucha treatment also induced weight loss and increased levels of liver SOD. Kombucha improved lipid profiles: reducing total cholesterol, TG, LDL, and increasing HDL levels compared with CFED and normal groups. This Kombucha has good potential as a functional beverage with anti-obese and lipid improving activity.
				In vivo assay	- n = 40 albino Swiss mice (*Mus musculus*) (4 groups of 10); - SOD activity; - Lipid profile (LDL, HDL, TG and total cholesterol); - Biomedical analysis of blood sample.	
[64]	To characterize the changes in physicochemical and microbiological composition, and in the phenolic profile of Kombucha during fermentation.	Black tea	25 °C 10 days	Characterization, and in vitro assay	- pH and total acidicity; - MC; - Theaflavin and thearubigin profile; - TPC; - antiplasmidial test.	Changes in the Kombucha phenolic profile take place during fermentation, which may lead to the higher bioctive potential and contribute to a better understanding of the Kombucha fermentation process.
[65]	To develop and evaluate the protective effect of blueberry fermented and unfermented Kombucha beverages after induction of gastric ulcer in mice.	Commercial Kombucha from Kombucha Caseira^®^	Not reported	Characterization	- pH, density and turbidity; - TPC; - Tannin and anthocyanin content; - AOA.	The use of blueberry for fermentation of Kombucha was favorable in relation to the physicochemical aspects: increased TPC, tannins and anthocyanins contents. However, the fermentation product had lower antioxidant activity when compared with the unfermented beverage. The gastroprotective effect of the fermented Kombucha beverage: the biochemical parameters remained at normal levels; however, some animals showed gastric lesions in an expressive area.
				In vivo assays	- n = 24 (4 groups (n = 6)) Swiss mice; - Biochemical analysis; - Lipid peroxidation and advance oxidation protein products determination.	
[66]	To incorporate butterfly pea into an innovative drink through a SCOBY fermentation and to evaluate the biological activity (in vitro and in vivo).	Butterfly pea flower Kombucha (KBPF)	20–25 °C 12 days	Characterization and in vitro assay	- Metabolomic profiling untargeted; - Lipase inhibition assay; - α-Glucosidase and α-Amylase inhibition assay; - AOA.	A total of 79 secondary metabolite compounds were successfully identified in KBPF. In vitro studies showed the potential activity of KBPF in inhibiting not only ABTS, but also lipid (lipase) and carbohydrate (α-amylase, α-glucosidase) hydrolyzing enzymes. In the in vivo study, the administration of KBPF (130 mg/kg BW) significantly alleviated metabolic disorders caused caused by a high-fat diet. Lipid profile (HDL, LDL, TC, TG), blood glucose, markers of oxidative stress, metabolic enzymes (lipase, amylase), and markers of inflammation (PGC-1α, TNF-α, and IL-10) were, in most cases, restored to normal values. The gut microbiota community analysis showed that KBPF has a positive effect on both the Bacteroidetes phylum and the Firmicutes phylum.
				In vivo assay	- n = 40 albino Swiss (*Mus musculus*) mice; - Metabolic and inflammatory biomarkers. - Study design of treatments; - Biomedical analysis of collected blood samples; - Gut microbiota community.	
[67]	To explore the effects of both temperature and time on the dynamic changes induced during fermentation on the properties of laver Kombucha.	Dried laver (*Porphyra* *dentata*) sheets	25 or 30 °C 22 days	Characterization	- pH and TA; - RS; - TAA; - TSS; - organic acid content; - MC; - Total of bioactive components; - AOA; - Sensory analysis.	Longer fermentation can promote the excessive accumulation of acid and can affect the taste of the final products. Higher fermentation temperatures expedite the fermentation process, enhancing the microorganism growth and biofilm yield with respect to the higher acidity and lower pH. Fermentation of laver Kombucha at 25 °C can maintain important total flavonoid compounds and enhance the α-amylase inhibitory activity.
[68]	To determinate of the chemical composition, microbiological SCOBY composition, mineral content, and organoleptic composition of new kinds of homemade Kombucha beverages using alternative kinds of sugars.	Black, green, and white teas (*Camellia sinensis*), from Kenya, Tanzania, and China, sugars (cane and coconut sugars)	22 °C 14 days	Characterization	- pH; - Alcohol, sugar, mineral contents; - Polyphenols profile; - ABA; - Identification of Kombucha microflor;a - Organoleptic evaluation.	Black and green tea beverages showed the highest antibacterial activity. The bacteria *E. coli* and *Salmonella* sp. were the most sensitive to the effects of Kombucha. A total of 17 bioactive compounds were found in Kombucha. Kombucha contained many elements such as aluminium, calcium, iron, potassium, magnesium, sodium, phosphorus, and sulphur. Tea mushroom microflora contained the following microorganisms: *Gluconacetobacter xylinus*, *Acetobacter xylinum*, *Bacterium gluconicum*, *Gluconobacter oxydans*, *Leuconostoc mesenteroides*, *Propionibacterium* spp., *Acetobacter nitrogenifigens*, *Gluconacetobacter kombucha*, *Saccharomyces cerevisiae*, *Candida vini*, *Schizosaccharomyces pombe*, *Pichia membranefaciens*, *Kloeckera apiculate*, *Kluyveromyces marxianus*, and *Pichia kluyveri*. The aroma most highly rated by the evaluators was that of Kombucha No. 4 from green tea and coconut sugar, while Kombucha No. 1 from black tea and cane sugar had a less desirable aroma.
[69]	To analyze the several preparation procedures in relation to the water-holding and oil-holding capabilities of hydrolysates made from Kombucha cellulose.	Not reported	25 °C 15 days	Characterization and in vitro assay	- Cellulose purification; - Cellulose hydrolysates produced. - Determination of oil and water-holding capacity; - Particle size; - Deep-fried donut production; - Water and oils contents - Texture profile analysis.	The water-holding capacity of the Kombucha cellulose hydrolysates was higher than for the intact Kombucha cellulose, while the oil-holding capacity was lower. The hydrolysates of Kombucha cellulose and the intact Kombucha cellulose were used to make deep-fried donuts. In vitro digestion results suggested that there would be no adverse health effects from substituting Kombucha cellulose hydrolysates into the deep-fried donut formula.
2021						
[70]	To evaluate the impact of the addition of pitanga and umbu-cajá fruits on the physicochemical parameters, volatiles, phenolics and antioxidant capacity of Kombucha.	Green tea and fruits: pitanga (*Eugenia uniflora* L.), and umbu-cajá, (*Spondia tuberosa*).	25 °C 48 h	Characterization and in vitro assay	- pH and TA; - TSS and TPC; - Profile of organic acids and sugars; - Profile of volatile compounds; - AOA; - Digestion.	After a simulated gastrointestinal digestion, the phenolic contents in all Kombuchas decreased, resulting in a significant drop in the antioxidant capacity. The findings demonstrated that pitanga and umbu-cajá contribute to diversifying and improving the chemical and bioactive characteristics of the Kombucha, resulting in a sweeter beverage, with a tendency to fruity aromas.
[71]	To investigate the effects of Kombucha tea based on blood glucose levels, total cholesterol, and PGC-1α in Swiss albino mice that were given CFED.	Seagrapes *(Caulerpa racemosa)*		In vitro assays	- Anti-glycation activity; - Tyrosinase inhibitory activity; - α-glucosidase inhibitory activity; - α-Amylase activity assay;	This Kombucha was shown to improve blood glucose level, total cholesterol level, and PGC-1α on mice fed with CFED. Anti-glycation, tyrosinase inhibition, α-glucosidase, α-amylase inhibition properties of Kombucha tea were also observed.
				In vivo assays	- n = 40 Swiss albino mice (n = 10 per group); - Blood glucose levels, PGC-1α; - Total cholesterol.	
[72]	To isolate, identify and characterize the microorganisms found in the Kombucha starter.	Papaya (*Carica papaya Linn*.)	37 °C 4 days	Characterization	- pH; - TSS; - Ethanol and acetic acid analysis; - Phylogenetic analysis.	These preliminary results showed the potential of using these isolated strains as starter cultures for the production of novel functional fermented beverages from papaya pulp and leaves.
[73]	To produce unfermented and Kombucha beverages, and assess their physicochemical characteristics, in vivo toxicities, antioxidant activities and antimicrobial properties.	*Malvaviscus arboreus* and *Camellia sinensis*	24 °C 14 days	Characterization	- pH and total acidity; - TSS; - TPC; - AOA; - MC and AMA.	It was possible to elaborate a fermented beverage using a non-conventional edible plant. Beverages produced with *Malvaviscus* showed antioxidant activity but not antimicrobial activity. Beverages produced with green tea showed high antioxidant and antimicrobial activity. First time the use of this in vivo model of toxicity.
				In vivo assay	- n = 50 *Galleria mellonella* larvae model (5 groups of 10 larvae); - Toxicity assay.	
[74]	To evaluate the effects of supplementation with Kombucha and green banana flour on Wistar rats fed with a cafeteria diet.	Green tea	Room temperature 15 days	In vivo assay	- n = 35 Wistar rats (5 groups); - Biochemical parameters; - Liver enzymes and histology; - Body composition (fat, protein, ash, and moisture levels of the viscerated carcasses); - Serum and liver AOA.	The intake of caffeine altered the lipid and liver profile of the animals and the consumption of Kombucha and green banana flour did not prevent these changes. The high polyphenols level in Kombucha did not exert a hepatoprotective effect as an antioxidant.
[75]	To develop a novel functional Kombucha beverage using laver, with beneficial physicochemical characteristics, antioxidant effects, and nutraceutical properties.	Dried laver (*Porphyra* *dentata*) sheets, dried green tea, black tea	25 °C 14 days	Characterization	- pH and TA; - RS; - MC; - Biofilm yield, colour, and TSS; - Organic acid content; - Bioactive components analysis; - AOA; - Sensory evaluation.	Tea Kombucha showed higher amounts of total phenols and flavonoids, and ferric-reducing antioxidant power, while ultrasound-assisted extracts exhibited the highest content of organic acid, especially, α- ketoglutaric, and acetic acid, which had the highest titratable acidity, lower pH value and enhanced antioxidant scavenging ability.
[76]	To develop and characterize a novel Kombucha prepared form medicinal mushrooms.	Mushrooms: *Coriolus versicolor* and *Lentinus edodes*	24 °C 11 days	Characterization and in vitro assay	- pH and total acids; - Total microorganisms; - Sucrose, glucose, fructose and ethanol contents. - Qualitative and quantitative chemical analysis of polysaccharides; - Cyotoxicity (PBMC cells); - Cytokines detection.	There is great potential for using medicinal mushrooms as a substrate in Kombucha fermentation. Hot water extraction of produced mushroom fruiting bodies enabled the generation of substrate, which stimulated total acid production and consequently provided shorter fermentation time. The Kombucha products possess significant amounts of bioactive components. The analysis of the biological properties of Kombucha polysaccharides suggests they have highly desirable immunomodulatory properties in human cell cultures.
[77]	To evaluate the natural potential of combination therapy of this natural product with doxorubicin as a chemotherapeutic agent.	2 Kombucha (pasteurized fermented green tea)	2 weeks and 3 weeks	In vitro assay	- Cyotoxicity; - Anticancer activity; - Apoptosis assays (HCT-116 cell line).	Kombucha beverage inhibited antiapoptotic function Kombucha beverages prepared from green tea possess interesting antiproliferative properties associated with significant antiapoptotic activity in cellular and molecular scales.
[78]	To study the effects of tea leaves on the antioxidant capacity, phenolic content, and bioaccessibility of Kombucha teas during fermentation, investigated by the simulated in vitro gastrintestinal digestion method.	5 teas: White tea (Silver Needle, China), green tea (Matcha, Japan), oolong tea (Milk Oolong, Taiwan), black tea (Keemun Black, China) and pu-erh tea (Golden pu-erh, China)	30 °C 15 days	In vitro assay	- Digestion enzymatic extraction; - TPC; - AOA; - Bioaccessibility.	Kombucha fermentation with tea leaves led to an increase in antioxidant capacity, TPC and bioaccessibility. Among the kombucha tea types, the green tea Kombucha had the highest antioxidant capacity levels. The bioaccessibility levels did not significantly change during the fermentation. The TPC content in the extractable fractions was higher compared with hydrolysable fractions due to complex interactions among the constituents.
[79]	To evaluate the effects of long-term storage at 4 °C on the pH, total phenolic and flavonoid contents, and free radical scavenging properties of Kombucha during 9 months with a sampling interval of 30 days, aiming to determine the period during which these parameters are stable.	Not reported	30 days	Characterization	- Monitoring of pH; - TPC and TFC; - AOA.	After 4 months, the phenolic content decreased significantly from the initial value, as well its antioxidant capacity. The pH value increased from 2.82 to 3.16. The novel findings of this pilot study revealed that Kombucha from sugared black tea can be stored at refrigerator temperature for four months. After this period the antioxidant properties of Kombucha are no longer retained.
[80]	To investigate the possibility of the fortification of traditional Kombucha beverage with different medicinal plant infusions.	Linden, lemon balm, sage, echinacea, mint, and cinnamon infusions	28 °C 14 days	Characterization and in vitro assay	- TPC; - AOA. - Organic acids and minerals contents; - Sensory analysis; - Gastrointestinal digestion assay; - Microbiological analysis.	After fermentation, the antioxidant capacity of the Kombucha increased. On days 0 and 9 of storage, the bioaccessibility of the total phenolics and antioxidant capacity in all of the samples showed a significant increase after gastric and intestinal digestion when compared with pregastric digestion. The antioxidant capacity after in vitro digestion at the beginning and end of storage in all of the beverages also increased after gastric digestion compared with pregastric digestion; however, it decreased after intestinal digestion.
[81]	To characterize fermented foods produced at small scale, archaeal and bacterial microbiota composition were determined and a preliminary in vitro gut microbiota experiment was conducted to assess the potential prebiotic or probiotic properties of the kraut product and Kombucha.	Black tea	Room temperature 7 days	In vitro assay	- Fermentation study; - Microbial DNA extraction; - Dual-index bacterial amplicon library preparation and sequencing.	The Kombucha fermentation was dominated by Gluconacetobacter, but also characterized by a high abundance of Bacteroides. The microbiota composition and dynamics were very different between the two Kombucha batches tested, suggesting redundancy in microorganisms’ fermentative roles. A preliminary in vitro fermentation study was indicative of a potential bifidogenic effect of microbial metabolites from Kombucha.
[82]	To characterize yerba-maté Kombucha (YMK) by investigating its oxidative stress inhibitory property with *Saccharomyces cerevisiae* yeast as the model and to determine its antibacterial activity against *Staphylococcus aureus* and *Escherichia coli*.	Yerba-maté	25 °C 12 days	Characterization	- pH and acidity; - RS; - TPC; - AOA and ABA.	The antioxidant activity was higher than that of the unfermented extracts. YMK exhibited antibacterial activity and was also effective at preventing the oxidative stress in *S. cerevisiae.* YMK contains bioactive compounds that have potential applications in the food industry. Polyphenols are the main compounds found in YMK, whose properties demonstrated in in vitro and in vivo tests, such as antioxidant activity, are potentially beneficial to health. Organic acids produced during fermentation inhibited bacterial growth, increasing the shelf life of the beverage.
2020						
[83]	To investigate the fermentation kinetics, metabolite production, microbiome and potential health promoting properties of 3 different Kombucha consortia.	Black tea	25 °C 15 days	Characterization and in vitro assays	- Metagenomic analysis; - Sugars, ethanol and acetic acid quantification; - Phenolic and aromatic compounds determination. - AOA and TPC; - AIA and antiprolifeartion evaluation (cancer cells).	Metagenomic DNA from the solid and liquid phases of 3 Kombucha consortia revealed differences across Kombuchas and between the two phases. Fermentation kinetics showed an association between the microbiota, sugar consumption, and secondary metabolite production. The most abundant species of bacteria were the same in all the samples, differing only in relative abundance. The yeast populations differed most considerably. Microbiota differences were observed in the biological profiles of the obtained teas.
[84]	To evaluate the antimicrobial activity of green tea Kombucha at two fermentation time points against *Alicyclobacillus* spp.	Green tea (local grower in Maringá, Paraná)	Room temperature 7 and 14 days	Characterization	- AOA; - AMA; - Compounds characterization;	Kombuchas fermented for different times showed effective inhibitory and bactericidal activities at low concentrations against all evaluated *Alicyclobacillus* spp. The length of fermentation affects the antimicrobial activity of Kombucha against spoilage bacteria: the longer the fermentation time, the greater the antimicrobial activity. Fermented green tea showed a higher number of metabolites than green tea not fermented by symbiotic bacteria.
[85]	To isolate and identify a yeast strain from Kombucha and evaluate in vitro its potential as a novel starter in beverage fermentation.	Black tea	30 °C 48 h	Characterization, and in vitro assay	- Yeast isolation, identification and DNA sequencing; - Phenotypic characterization (sugar, carbohydrates, cholesterol, and pH); - Fermentation properties (pH, high-sucrose stress tolerance, organic acids content, AOA, TPC and TFC); - Digestion.	The yeast strain has a cholesterol-lowering capacity of 45%, grew at a temperature of 37 °C and is resistant to pH 1.5. The yeast has pH reduction capacity and can produce organic acids and volatile compounds such as 2-phenylethanol. The fermented beverage also has high total phenolics and flanonoids content and showed great AOA and AMA. The findings of this research provide strong evidence that *Starmerella davenportii* Do18 has good fermentation properties and is a potential starter in food and beverage fermentation.
[86]	To determine the microbiota properties of Kombucha and to isolate the active bacteria and/or yeasts in aflatoxin B_1_ (AFB1) biodegradation. To select and identify strains involved in AFB1 degradation. To evaluate the byproducts’ safety by cell cytotoxicity tests.	Black tea	25 °C 7 days	Characterization and in vitro assay	- MC; - Degradation and absortion of mycotoxin content; - Cytotoxicity (Hep2 cells).	After 7 days of fermentation, Kombucha was able to degrade 97% of AFB1 in black tea. Yeasts present in Kombucha: *Pichia occidentalis*, *Candida sorboxylosa* and *Hanseniaspora opuntiae*. The highest AFB1 degradation capacity was accorded to *P. occidentalis* (59%) when cultivated in black tea. Cytotoxicity tests showed that the biodegraded products were less toxic than pure AFB1.
[87]	To investigated the anti-virulence activity of a polyphenolic fraction previously isolated from Kombucha.	Black tea	28 °C 14 days	Characterization	- ABA; - Effect on bacterial motility and proteolytic activity;	The overall results imply that Kombucha might be considered as a potential alternative source of anti-virulence polyphenols against *V. cholerae*. This is the first report on the anti-virulence activity of Kombucha, mostly attributed to its polyphenolic content.
[88]	To determine the antioxidant capacities, and antibacterial and antiproliferative activities.	Green tea or black tea	25 °C 10 days	Characterization and in vitro assay	- pH and total acidity; - RS, organic acids and ethanol; - MC; - TPC; - Theaflavin and thearubigin; - AOA and ABA; - Cytotoxicity and proliferation (A549, HCT8, CACO-2, IMR90 cells).	A greater diversity and abundance of phenolic compounds were detected in black tea Kombucha, which resulted in a higher antioxidant capacity. The green tea Kombucha was the only one that presented antibacterial activity against all the bacteria tested and an increased antiproliferative activity against the cancer cell lines. The type of tea used in the Kombucha production influences its bioactive composition and properties.
[89]	To evaluate the characteristics and the antidiabetic potential of Kombucha herbal tea from *R. mucronata* fruit based on in vitro, chemical, and physical analyses.	Mangrove fruit herbal tea was made from *R. mucronata* fruit.	Room temperature 7, 14, and 21 days	Characterization	- Antidiabetic activity assay; - TPC; - Total acid analysis; - Organoleptic test.	The sugar concentration and fermentation time significantly affected the characteristics of the produced Kombucha in inhibiting α-glucosidase. The optimum treatment for inhibition was at 10% sugar concentration- The Kombucha from *R. mucronata* fruit had a pH of 3.11 and contained total phenolics of 19,679.82 mg GAE/100 g, 0.52% of total acids, and was quite preferred by panelists.
[90]	To present a comprehensive evaluation of the ferments obtained from green coffee beans after different fermentation times with Kombucha.	Arabica green coffee beans	25 °C 7, 14 and 28 days	Characterization and in vitro assay	- Determination of bioactive compounds; - AOA; - OS and SOD; - Cytotoxicity; - Metallopeptidases inhibition; - Transepidermal water loss and skin moisture; - Determination of sun protection factor (in vitro).	Results for the ferments were compared with the green coffee extract that was not fermented. The fermentation time has a positive effect on the content of bioactive compounds and antioxidant properties. The highest values were recorded for the tested samples after 28 days of fermentation. After 14 days of the fermentation process, it was observed that the analyzed ferments were characterized by low cytotoxicity to keratinocytes and fibroblasts. On the other hand, the short fermentation time of 7 days had a negative effect on the properties of the analyzed ferments.
2019						
[91]	To compare the antidiabetic activity of snake fruit Kombucha, black tea Kombucha and metformin in streptozotocin-induced diabetic rats.	Black tea	Room temperature 14 days	Characterization	- pH and total acidity; - RS and AOA; - TPC and TSS; -Tannins.	The 3 treatments (snake fruit Kombucha, black tea Kombucha and metformin) were effective as diabetes therapy agents in the rat model by lowering FPG, improving oxidation stress status and lipid profiles. Improvements in the pancreas by these 3 treatments. The snake fruit Kombucha was as effective as the metformin in managing the induced diabetes, and more effective than the black tea Kombucha.
				In vivo assay	- n = 25 Wistar rats (5 groups) - FPG, SOD-malondialdehyde levels; - Lipid profiles; - Pancreas immunohistochemistry staining.	
[92]	To investigate the feasibility of transforming soy whey into a novel functional beverage using Kombucha consortium.	“Commercial Kombucha”	28 °C 7 days	Characterization	- pH and TA; - RS; - TFC; - Glucuronic acid, organic acids and isoflavones contents; - AOA and AMA; - Sensory analysis.	The antioxidant capacity of kombucha-fermented soy whey was significantly enhanced and showed antibacterial activity against *Staphylococcus aureus*, *Bacillus subtilis* and *Escherichia coli*. The fermentation produced new aroma-active volatiles (esters and higher aldehydes) which imparted the fruity flavor of soy whey and improved its sensory quality.
[93]	To provide a deep insight into traditionally prepared Kombuchas and gain new knowledge on the use of rooibos herbal tea, which has never been considered as a substrate, in spite of its well-known bioactivity and potential health benefits.	Infusion teas: green tea “Sencha”, black tea “Ceylon”, and, rooibos (*Aspalathus linearis*)	27 °C 14 days	Characterization and in vitro assay	- Sugar and acid organics analysis; - TPC; - AOA; - Catechins identification and quantification; - Oxidative cell treatments and citotoxicity (L929); - Microbiology; - Molecular identification of acetic acid bacteria and yeast isolates.	All of the Kombuchas showed similarity in bacterial composition, with the dominance of *Komagataeibacter* spp. The yeast community was significantly different among all tea substrates, between 7 and 14 days of fermentation and between the biofilm and Kombucha, indicating the influence of the substrate on the fermenting microbiota. Kombucha from rooibos has a low ethanol concentration, and a glucuronic acid level comparable to black tea. Although antioxidant activity was higher in black and green kombucha compared with rooibos, the latter showed an important effect on the recovery of oxidative damage on fibroblast cell lines against oxidative stress.
[94]	To evaluate if black goji berry and red goji berry fruits are suitable for Kombucha beverage production.	Black tea, red goji berry (*Lycium barbarum* L.) and black goji berry (*Lycium ruthenicum* Murr.)	28 °C 48 h	Characterization and in vitro assay	- pH and total acidity; - Determination of water soluble solids. - TPC; - AOA; - Sensory analysis; - Digestion.	Total phenolic content of all Kombucha samples fluctuated during fermentation and storage. All Kombucha samples had higher antioxidant activity than their infusions. Total phenolic content and antioxidant capacity in in vitro predigestion and postdigestion of samples were ranked as follows: black tea Kombucha > black goji berry Kombucha > red goji berry Kombucha.
2018						
[21]	To investigate the possibility of producing of a new variety of Kombucha beverages.	Yarrow (*Achillea millefolium* L.) (Southeast region of Serbia)	25 °C 7 days	Characterization and in vitro assay	- pH and total acidity; - Biomass measurement; - Organic acids analysis; - TPC and TFC; - vitamin C analysis; - AOA; - Sensory analysis; - Cytotoxity (RD, Hep2c, L2OB).	Kombucha beverages were successfully produced on yarrow infusions and subcritical water extracts. The most suitable substrate was obtained with the following fermentation process parameters: subcritical water extract at 115 °C, and 2.26 g of yarrow flowers in 500 mL water. Organic acids (oxalic, formic, acetic, succinic and malic) content was higher in beverages produced from subcritical water extracts. Vitamin C values were higher in beverages produced with infusions. Total phenols and flavonoids contents depended directly on the amount of herb. Yarrow kombucha produced by fermentation on subcritical water extracts showed higher antioxidant activity but lower antimicrobial and antiproliferative activity in comparison with products obtained by infusions. Kombucha beverages produced from subcritical water extracts of yarrow had the highest sensory score.
[95]	To investigate fermentation of snake fruit with the Kombucha consortium. To evaluate the physicochemical and sensory properties of the fermented products. To assess the antioxidant and antibacterial activities of the most promising cultivars and their bioactive compounds.	“Commercial Kombucha”	Room temperature 14 days	Characterization	- Physicochemical analysis; - MC; - AOA and ABA; - Bioactive compounds analysis; - Sensory analysis.	Sugared snake fruit juices from five Indonesian snake fruit cultivars (*Salak Doyong*, *Salak Madu*, *Salak Pondoh*, *Salak Segaran*, and *Salak Suwaru*) can potentially be used to produce fermented beverages with the Kombucha consortium. The fermentation affected the physicochemical and sensory properties of the juices, enhanced the antioxidant and antibacterial activities of the resulting products, and increased the levels of the bioactive compounds, indicating the desirable overall properties of the fermented beverage.
[96]	To enhance the health-related benefits of pollen by fermentation with a Kombucha/SCOBY consortium.	Green tea (Basilur green tea, Ceylon)	28 °C 30 days	Characterization	- Total polyphenols; - TPC and TFC; - AOA; - Lactic acid; - Structural and morphological analysis; - Real-time PCR technique; - Analysis of organic acids, hydroxy-acids and short chains fatty acids; - Biocompatibility (normal mouse fibroblast cell line); - Antitumoral activity (Hep-2, Caco-2 cells).	The pollen addition increased the proportion of Lactic acid bacteria in the total population of SCOBY microbial strains. Scanning electron microscopy images highlighted the adhesion of the SCOBY bacteria to pollen. The content of bioactive compounds (polyphenols, soluble silicon species and short chain fatty acids) was higher in the fermented pollen and the product shows a moderate antitumoral effect on Caco-2 cells. The health benefits of pollen are enhanced by fermentation with a Kombucha consortium.
2017						
[97]	To investigate the antioxidant activity and anti-inflammatory effects of Kombucha analogues from oak by examining their modulation ability on macrophage-derived TNF-alpha and IL-6.	Herbal infusions from oak and black tea	28 °C 7 days	Characterization and in vitro assay	- Chemical characterization; - Sugar, gluconic and glucuronic acid content; - TPC; - AOA and AIA; - Cell viability (THP-1) - Suppression of TNF-alpha and IL-6 release; - Nitric oxide and OS determination.	Kombucha analogues from oak showed good antioxidant properties, attributed particularly to their phenolic composition. The major effect detected was their ability to suppress lipopolysaccharide-induced production of nitric oxide, TNF-alpha and IL-6, showing an important anti-inflammatory activity.
2016						
[98]	To investigate the antifungal activity of Kombucha tea ethyl acetate fraction (KEAF) against Malassezia species obtained from the patients with seborrheic dermatitis.	Kombucha tea, black tea (Golestan, Tehran, Iran)	24 °C 14 days	In vitro assay	- Culture and identification of *Malassezia* species; - DNA sequencing; - Antifungal properties.	The results of the DNA sequence analysis indicated that *M. furfur* was the predominant species, followed by *M. globosa*, *M. sloofie*, *M. sympodialis*, and *M. restricta*, respectively. The findings of the study highlight the antifungal properties of KEAF. KEAF showed inhibitory activity against *Malassezia* species. KEAF had the lowest and highest MIC value against *M. sloofie* and *M. restricta*, respectively.
2015						
[99]	To assess changes in Kombucha’s antioxidant activity and phenolic compounds during fermentation as affected by different ratios of sugared black tea decoction and wheatgrass juice (WGJ).	Sweetened black tea and WGJ (*Triticum aestivum* L.)	29 °C 12 days	Characterization	- pH; - TPC and TFC; - Total anthocyanin content; - Phenolic composition; - AOA.	The results showed that the TPC, TFC and AOA of the modified Kombucha were higher than those of traditional preparations. All WGJ-blended Kombucha preparations were characterized as having higher concentrations of various phenolic compounds such as galic acid, catechin, caffeic acid, ferulic acid, rutin, and chlorogenic acid, compared with traditional preparations. The highest antioxidant activity was obtained using a 1:1 (*v*/*v*) black tea decoction to WGJ ratio and 3 days of fermentation, which produced various types of phenolic acids.
2014						
[100]	To investigate the effects of Kombucha inoculum as a new starter culture for milk fermentation, during 14 days of storage.	Black tea (*Camellia sinensis)*	25 °C 7 days	Characterization	- pH; - Protein total; - Degree of proteolysis; - ACE inhibitory activity; - AOA; - Vitamin C content; - Sensory analysis.	The Kombucha fermented milk product showed similar trend of changes in pH, degree of proteolysis, and sensory properties as products obtained by probiotic and yoghurt starters. Significant ACE inhibitory was determined in all fermented products, which increased during storage, and the Kombucha product had the highest ACE activity at the end of storage compared with probiotic and yoghurt products. In all products, higher radical scavenging activity was determined, while both activities slightly decreased during storage.
2013						
[101]	To investigat the antidiabetic and antioxidant effects of Kombucha in comparison with unfermented black tea, in alloxan monohydrate (ALX)-induced diabetic rats.	Black tea	Room temperature 14 days	Characterization	- TPC and TFC; - Organic acids composition; - AOA.	ALX exposure lowered the body weight and plasma insulin by about 28.12% and 61.34%, respectively, and elevated blood glucose level and glycated Hb. The oxidative stress-related parameters, including lipid peroxidation end products (increased), protein carbonyl content (increased) and glutathione content (decreased), and antioxidant enzyme activities, were also altered in the pancreatic, hepatic, renal and cardiac tissues of diabetic animals. The results showed the significant antidiabetic potential of the fermented beverage as it effectively restored ALX-induced pathophysiological changes. It could ameliorate DNA fragmentation and caspase-3 activation in the pancreatic tissue of diabetic rats. Although unfermented black tea is effective in the above pathophysiology, Kombucha tea was found to be more efficient. This might be due to the formation of some antioxidant molecules during the fermentation period.
				In vivo assay	- n = 36 Swiss albino rats (6 groups); - Tissue collection; - Fasting serum glucose level; - Glycosylated hemoglobin and insulin; - Biochemical parameters; - Histological studies; - Measurment of tissue ROS levels; - Estimation of lipid and protein damage. - Antioxidant enzymes; - Glutathione level DNA fragmentation assay.	
[102]	To examine the anti-angiogenic effect of Kombucha on angiogenesis stimulators/regulators in a human androgen-independent prostate cancer cell line.	Kombucha baby mat with its mother liquor “commercial”	28 °C 14 days	In vitro assay	- Cell viability (PC-3 cells); - RNA isolation; - Gene expression.	Kombucha significantly inhibits angiogenesis through alterations in the expression of angiogenic stimulators. Kombucha drink may lead to growth inhibition of tumors and a reduced likelihood of cancer metastasis with daily limited consumption. This suggests the need for clinical investigation of Kombucha in the prevention of cancer invasion.
2012						
[103]	To investigate and compare the hypoglycemic and antilipidemic effects of Kombucha and black teas, two natural drinks commonly consumed around the world, in surviving diabetic rats.	Black tea powder (Lipton)	28 °C 12 days	In vivo asssay	- n = 48 (6 groups); - Histologcal assays; - Plasma and pancreas ɑ-amylase activity, glucose levels; - pancreas lipase activity and plasma lipids concentrations; - liver–kidney dysfunction indices.	Compared with black tea, Kombucha tea was a better inhibitor of α-amylase and lipase activities in the plasma and pancreas and a better suppressor of increased blood glucose levels. Kombucha was noted to induce a marked delay in the absorption of LDL-cholesterol and triglycerides and a significant increase in HDL-cholesterol. Histological analyses showed an ameliorative action on the pancreas and effective protection of liver–kidney function in diabetic rats, evidenced by significant decreases in aspartate transaminase, alanine transaminase, and gamma-glytamyl transpeptidase activities in the plasma, as well as in the creatinine and urea contents.
2008						
[104]	To elucidate the relationship between the fermentation time and antioxidant activities of Kombucha. To determine changes in free-radical scavenging abilities along with pH and total phenolic compounds of Kombucha tea during fermentation.	Green tea, and black tea, and tea waste material. From *Camellia sinensis* (L).	24 °C 18 days	Characterization	- pH; - TPC; - AOA; - Anti-lipid peroxidation.	The prepared Kombucha teas have excellent antioxidant activities. Kombucha exhibited increased free-radical scavenging activities during fermentation. The extent of these activities depends upon the fermentation time, type of tea material and the normal microbiota of the Kombucha culture, which in turn determined the forms of their metabolites.
2006						
[105]	To study the effects of the origins of Kombucha on antioxidant ability of Kombucha collected from households throughout Taiwan during fermentation.	Black tea leaf (Ten-Ren, Taiwan)	30 °C 15 days		- TPC; - AOA. - Inhibition of linoleic acid peroxidation measure.	The Kombucha exhibited increased antioxidant activities during fermentation. The extent of activity depended on the culture period and starter origins, which in turn determined the forms of their metabolites.

Abbreviations: TPC (total phenolic content), TFC (total flavonoid content), AOA (antioxidant activity), AMA (antimicrobial activity), TSS (total soluble solids), RS (determination of reducing sugars), ABA (antibacterial activity), AFA (antifungal activity), GBK (ginseng berry Kombucha), HepG2 (human liver cell line), OS (oxidative stress), GI (glycemic index), II (insulin index), BMI (body mass index), EIC (enzymatic inhibition capacity), KBT (Kombucha black tea), KGT (Kombucha green tea), SOD (superoxide dismutase), LDL (low-density lipoprotein cholesterol), HDL (high-density lipoprotein), TG (triglycerides), CFED (cholesterol and fat-enriched diets), SCOBY (symbiotic culture of bacteria and yeast), MC (microbiological characterization), KBPF (butterfly pea flower Kombucha), TA (titratable acidity), TAA (total amino acid content), PBMC (human peripheral blood mononuclear cells line), HCT-116 (human colon cancer cell line), YMK (yerba-maté Kombucha), AIA (anti-inflammatory activity), AFB1 (aflatoxin B1), Hep2 (human epithelial type 2 cell line), A549 (lung adenocarcinoma epitelial cell line), HCT8 (ileocecal colorectal adenocarcinoma cell line), CACO-2 (colorectal adenocarcinoma epithelial cell line), IMR90 (normal lung cell), FPG (fasting plasma glucose), L929 (mouse fibroblasts cell line), RD (cell line derived from human rhabdomyosarcoma), Hep2c (cell line derived from human cervix carcinoma-HeLa derivative), RT-PCR (real-time reverse transcription polymerase chain reaction), THP-1 (human monocytic cells line), KEAF (Kombucha tea ethyl acetate fraction), WGJ (wheatgrass juice), ALX (alloxan monohydrate), ROS (reactive oxygen species), PC-3 (Human prostate cancer cell line).
